# Targeting the proline-glycine-proline-protease feed-forward loop attenuates primary graft dysfunction after lung transplantation

**DOI:** 10.3389/fimmu.2026.1655536

**Published:** 2026-02-20

**Authors:** Yasufumi Goda, Stefi Lee, Adya Chawda, Xin Xu, Mohd Moin Khan, Gary Visner Do, Emma Hills, Andres L Pena, Patricia D.C. Lopez, Amit Gaggar, Antonio Coppolino, Camila Hochman-Mendes, Gabriel Loor, Mudassir Meraj Banday, Nirmal S Sharma

**Affiliations:** 1 Division of Pulmonary and Critical Care Medicine, Brigham and Women’s Hospital, Harvard Medical School, Boston, MA, United States; 2 Baylor College of Medicine, Houston, TX, United States; 3 Veterans Affairs (VA) Boston Medical Center, West Roxbury Campus, Boston, MA, United States; 4 Division of Pulmonary and Critical Care Medicine, University of Alabama at Birmingham, Birmingham, AL, United States; 5 Boston Children’s Hospital, Boston, MA, United States; 6 The Texas Heart Institute, Houston, TX, United States

**Keywords:** ischemi-reperfusion injury, lung allograft injury, lung transplantation, extracellular matrix–derived peptides, primary graft dysfunction

## Abstract

**Introduction:**

Primary graft dysfunction (PGD) is the leading cause of early mortality after lung transplantation, yet no targeted therapy exists. We investigated whether the collagen-derived matrikine proline-glycine-proline (PGP) drives neutrophil-predominant injury in PGD and whether its neutralization confers protection.

**Methods:**

Human mini-bronchoalveolar lavage (BAL) fluid was collected 72 hours post-transplantation from recipients with grade 3 PGD and non-PGD controls. In parallel, a murine orthotopic lung transplantation model incorporating 18 hours of cold ischemia was used to reproduce PGD; mice received vehicle (PBS) or the PGP-sequestering tripeptide L-arginine-threonine-arginine (RTR) immediately before reperfusion. Histology, immunofluorescence, LC-MS/MS quantification of acetyl-PGP (acPGP), gelatin zymography for active MMP-9, and ELISA for MMP-9 and prolyl endopeptidase (PE) were performed four hours later.

**Results:**

Human PGD BAL contained approximately fourfold higher acPGP, along with significantly elevated MMP-9 and PE, compared with PGD 0 controls. Murine PGD allografts similarly demonstrated dense neutrophilic infiltrates and increased acPGP, MMP-9, and PE expression. RTR treatment markedly reduced histologic injury, neutrophil accumulation, and composite PGD scores while improving oxygenation and allograft lung function. RTR also restored acPGP, MMP-9, PE, and active MMP-9 levels to near-baseline compared with vehicle-treated PGD allografts.

**Conclusion:**

These findings delineate a feed-forward PGP-protease circuit linking extracellular matrix degradation to neutrophil recruitment and vascular leak. Neutralizing PGP effectively disrupts this circuit, attenuating graft injury. By connecting extracellular matrix-derived signals to innate immune activation, this work broadens the immunopathologic framework of PGD.

## Introduction

Primary graft dysfunction (PGD) is the leading cause of early allograft failure after lung transplantation, occurring in approximately 30% of recipients and markedly compromising long-term survival (1). Risk is magnified by donor factors—advanced age, active smoking, and prolonged cold-ischemic time—as well as recipient characteristics such as pulmonary arterial hypertension, idiopathic pulmonary fibrosis, and obesity (2, 3). Despite these well-defined predictors, management remains limited to supportive care and, in refractory cases, retransplantation; no pharmacologic strategy currently prevents or treats PGD, leaving a critical therapeutic void (4).

Clinically, PGD is defined by diffuse bilateral alveolar infiltrates and a PaO_2_/FiO_2_ ratio < 300 within 72 h of reperfusion (5). Histopathology reveals a pronounced neutrophilic infiltrate and microvascular hyperpermeability (6). Contemporary models attribute this phenotype to donor-derived oxidative injury of endothelial and epithelial cells, which initiates chemokine-driven recruitment of recipient neutrophils and lymphocytes (7). Our group has identified a central role for the matrikine proline-glycine-proline (PGP) in amplifying the neutrophil-dominant response and endothelial leak in acute lung injury and post-transplant cardiac rejection (8, 9). Generated from collagen degradation, PGP ligates CXCR2 on neutrophils, activates ERK1/2-MAPK signaling, and promotes release of matrix metalloproteinase-9 (MMP-9) (10). Together with prolyl endopeptidase, MMP-9 further degrades collagen and elastin, generating additional PGP and establishing a self-propagating cycle of extracellular matrix destruction and neutrophil recruitment that exacerbates lung injury and vascular leakage (11, 12). The complementary tripeptide L-arginine-threonine-arginine (RTR) sequesters acetylated PGP and attenuates neutrophilia, thereby providing pharmacologic tractability to this feedforward cycle of injury (13).

Accordingly, we hypothesize that neutrophil-driven extracellular-matrix degradation releases the matrikine proline-glycine-proline (PGP), which amplifies neutrophilic inflammation and microvascular leak, culminating in PGD. To test this hypothesis, we employed human specimens and a murine lung transplant PGD model with peptide-based inhibitors of the PGP axis. Our findings identify PGP-dependent signaling as a mechanistic driver of PGD and highlight this pathway as a tractable therapeutic target.

## Methods

### Collection of human specimens

Using an IRB-approved protocol (Mass General Brigham IRB#2022P000210, Baylor College of Medicine IRB#H-42256), mini-bronchoalveolar lavage (mini-BAL) samples were obtained from mechanically ventilated patients within 72 hours post-lung transplantation. PGD was adjudicated using established ISHLT guidelines (5). The demographic characteristics of the human study cohort are summarized in **Tables 1** and **2**. Briefly, mini-BAL was performed by administering 30 ml of sterile saline via the endotracheal tube, followed by immediate suctioning using an 8-Fr suction cannula. Mini-BAL was then centrifuged at 3,500 revolutions/min for 15 min, and the supernatant was stored at −80°C until analysis using published methodologies (9).

**Table 1:** Demographic characteristics of patients included in the PGP evaluation.

Age range	Gender	Pretx diagnosis	PGD (Y/N)	PGD grade	CPB time (minutes)	Total ischemic time (minutes)
60-69	F	COPD	Y	3	182	Right 235, Left211
60-69	F	ILD	Y	3	210	Right 278, Left 303
30-39	F	IPAH	Y	3	176	Right 276, Left 313
50-59	F	COPD	N	0	115	Right 227, Left 265
60-69	M	IPF	N	0	208	Right 248, Left 296
50-59	M	IPF	N	0	210	Right 264 Left 308
70-79	M	ILD	N	0	116	Right 202, Left 232

**Table 2:** Demographic and clinical characteristics of the validation cohort analyzed for MMP-9 and PE levels.

Age range	Gender	Pretx diagnosis	PGD (Y/N)	PGD grade	Age range	Gender	Pretx diagnosis	PGD (Y/N)	PGD grade
40-49	M	ILD	Y	3	18-29	F	CF	N	0
70-79	M	COPD	Y	3	30-39	M	CF	N	0
60-69	M	ILD	Y	3	70-79	M	ILD	N	0
60-69	M	COPD	Y	3	18-29	M	CF	N	0
60-69	F	IPF	Y	3	40-49	F	CF	N	0
60-69	M	IPF	Y	3	50-59	F	COPD	N	0
50-59	M	ILD	Y	3	60-69	F	COPD	N	0
60-69	M	ILD	Y	3	60-69	M	ILD	N	0
60-69	F	COPD	Y	3	50-59	M	ILD	N	0

COPD, chronic obstructive pulmonary disease; ILD, interstitial lung disease; IPAH, idiopathic pulmonary arterial hypertension; IPF, idiopathic pulmonary fibrosis; CF, Cystic fibrosis Pretx, Pretlansplant CPB, cardiopulmonary bypass.

### Murine PGD model

Under an IACUC-approved protocol, murine lung transplant surgery was performed (MGB IACUC No. 2022N000156). A murine model of primary graft dysfunction through single orthotopic lung transplantation after prolonged cold ischemia of 18 hours (OLT-PCI) was performed using established techniques (14, 15). There were four groups in the study: A) Control group: a seven- to nine-week-old male BALB/c donor left lung was implanted into a C57BL/6 male (The Jackson Laboratory) mouse immediately after procurement without any cold ischemia, B) PGD group: seven- to nine-week-old male BALB/c donor left lung was implanted into a C57BL/6 male (The Jackson Laboratory) mouse after 18 hours of cold ischemia (donor lungs wrapped in perfadex solution and kept in 4 degree Celsius. C) Vehicle PGD group: seven- to nine-week-old male BALB/c donor left lung was implanted into a C57BL/6 male (The Jackson Laboratory) mouse after 18 hours of cold ischemia (donor lungs wrapped in perfadex solution and kept in 4 degree Celsius. Mouse received 50 μL of phosphate-buffered saline (PBS)intravenously, via penile vein injection, prior to reperfusion, D) RTR PGD group: seven- to nine-week-old male BALB/c donor left lung was implanted into a C57BL/6 male (The Jackson Laboratory) mouse after 18 hours of cold ischemia (donor lungs wrapped in perfadex solution and kept in 4 degree Celsius. Mouse received RTR, 250 μg in 50 μL PBS intravenously, via penile vein injection, prior to reperfusion. All mice were sacrificed after 4 hours of reperfusion using humane techniques and bronchoalveolar lavage (BAL), blood and tissue specimens collected. Likewise, we conducted a similar PGD model using syngeneic (B6→B6) transplant with similar ischemic parameters and RTR/vehicle to evaluate the role of adaptive versus innate immunity in RTR mediated PGD attenuation.

### Assessment of oxygenation and lung function

During the assessment of oxygenation and lung function, the right pulmonary hilum, including the accessory lobe, was occluded with a vascular clamp under median sternotomy, and the left lung was ventilated with a tidal volume of 5 mL/kg and a respiratory rate of 120 breaths/min. Mice were mechanically ventilated using a volume-controlled ventilator (Rovent, Harvard Apparatus, Holliston, MA, USA). Dynamic compliance and inspiratory capacity were measured using the built-in lung mechanics module of the Rovent ventilator. The fraction of inspired oxygen was maintained at 1.0. Five minutes after occlusion of the right hilum, arterial blood gas parameters were analyzed using an i-STAT handheld analyzer (Abbott Laboratories, Abbott Park, IL, USA) according to the manufacturer’s instructions.

### Murine bronchoalveolar lavage analysis

Briefly, after anesthesia, the native right lung bronchus was clamped first, and then BAL was performed via a tracheal cannula using 0.7 mL of sterile PBS, which was instilled three times and suctioned back sequentially. BAL fluid was centrifuged at 1500 rpm for 10 minutes at 4°C. the supernatant was separated into aliquots and frozen at -80°C until analysis. The supernatant was then analyzed for PGP peptide levels. The remaining cell pellets from all the lavages were used for cell counts. Differential cell counting was performed on air-drive cytospin preparations stained by the Protocol HEMA3 stain set. At least 200 cells were counted and the absolute number of neutrophils was calculated.

### Histology and immunofluorescence

Upon euthanasia (CO_2_ inhalation with cervical dislocation as a secondary method), the lungs were removed and immersed in fresh fixative for at least 24 hours. Following paraffin embedding, sections were cut and stained with hematoxylin and eosin for histologic and morphometric analysis. Histological scores of acute lung injury were graded on a scale based on morphological appearance: normal (0%), mild (<10%), moderate (10–50%), or severe (>50%) abnormalities, corresponding to scores of 0, 1, 2, and 3, respectively (16). Five randomly selected alveolar areas at high-power fields (HPFs) were evaluated from each lung section, with a total of four sections analyzed per group (20 HPFs in total). The number of macrophages, was quantified using ImageJ2 software (NIH, Bethesda, MD, USA). Likewise, for immunofluorescence studies, the tissue sections were deparaffinized, rehydrated, and subjected to antigen retrieval. The sections were then blocked and incubated overnight at 4°C with primary antibodies. After washing, secondary antibodies were applied, and the slides were examined under a microscope. The complete immunofluorescence protocol, including details on the antibodies and detection methods, is provided in the **Supplementary Methods**.

### ELISA

For murine lung lysate analysis, one section of lung was collected, homogenized, and resuspended in PBS. Total MMP-9 and prolyl endopeptidase (PE) concentrations in the lung tissue lysates were quantified using commercially available ELISA kits according to the manufacturers’ protocols (MMP-9: R&D Systems; PE: MyBioSource). For human BAL samples, MMP-9 and PE levels were measured using human-specific ELISA kits from Abcam based on a sandwich ELISA format. Values below detection limits were excluded. Murine lung tissue was analyzed for inflammatory cytokines using a multiplex cytokine assay. Details in **Supplementary Methods**.

### Gelatin zymography

Lung tissue homogenates were prepared in lysis buffer without protease inhibitors and centrifuged at 12,000 × g for 10 min at 4°C. Equal amounts of protein (54 μg) were mixed with non-reducing sample buffer and loaded onto 10% SDS–polyacrylamide gels containing 0.1% gelatin (Sigma-Aldrich). After electrophoresis, gels were washed twice in 2.5% Triton X-100 for 30 min to remove SDS and renature the gelatinases. Gels were then incubated in enzyme activation buffer (50 mM Tris-HCl, pH 7.5, 5 mM CaCl_2_, 0.02% NaN_3_) at 37°C for 18 h. Following incubation, gels were stained with 0.5% Coomassie Brilliant Blue R-250 and destained until clear lytic bands appeared against a blue background. Gel images were captured, and band intensities corresponding to MMP-9 activity were quantified using ImageJ software (NIH, Bethesda, MD,USA).

### Mass spectrometry

PGP peptides in murine and human BAL samples were quantified using an MDS Sciex (Applied Biosystems) API-4000 triple-quadrupole mass spectrometer coupled with a Shimadzu high-performance liquid chromatography system and a 2.0 x 150 mm Jupiter 4u Proteo column (Phenomenex) [7].

### Statistical analyses

For statistical testing, normality for each data set was assessed by Shapiro-Wilk test followed by the appropriate statistical testing (*t* -test or non-parametric, 2-tailed, alpha cutoff for statistical significance was <0.05). data was presented as mean+/- SEM. Details of individual tests used are provided in the figure legends. GraphPad Prism version 10.0 was used to analyze and plot the data. Illustrations were generated using BioRender Illustrator.

## Results

### Neutrophil infiltration and PGP accumulation characterize PGD

**Figure 1:**
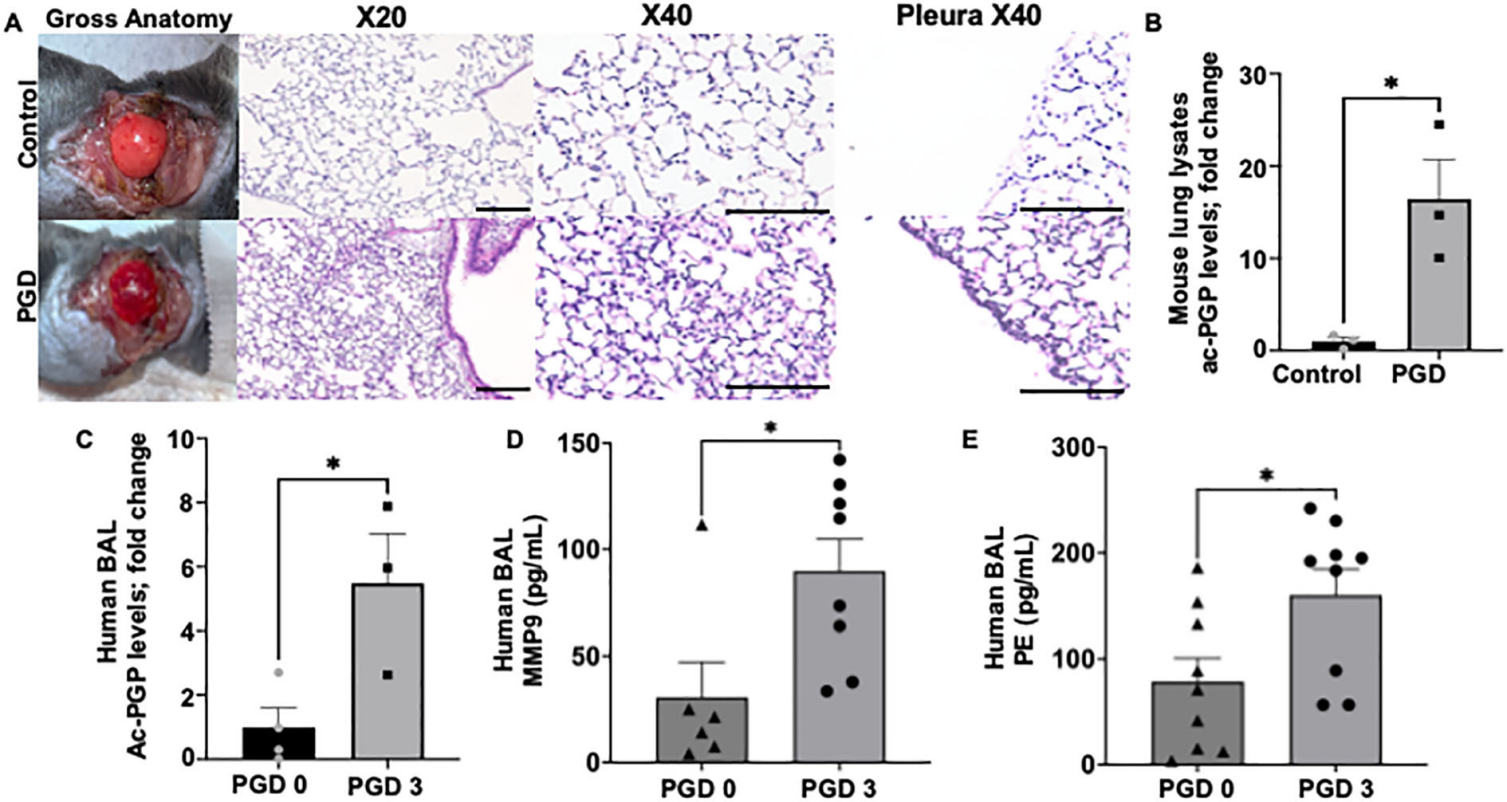
Neutrophil infiltration and PGP accumulation characterize experimental PGD. **(A)** representative gross anatomy and H&E-stained sections of control and PGD mouse allografts four hours after reperfusion. PGD lungs show interstitial and perivascular neutrophilic infiltrates, pleural thickening, and disrupted alveolar architecture. Scale bar = 100 µm. **(B)** Fold change quantification of bio-active acetyl-PGP (acPGP) in murine whole-lung lysates by LC–MS/MS. *P < 0.05 (unpaired *t*-test). **(C)** Fold change PGP concentrations measured in bronchoalveolar lavage (BAL) fluid from lung-transplant recipients with grade-3 PGD versus PGD 0 controls (72 hours). **(D, E)** Quantitative ELISA of MMP-9 **(D)** and PE **(E)** in human PGD0 and PGD3 patient samples. Both MMP-9 and PE activities were higher in PGD3 patients than in PGD0 patients. Sample size: n=6–9 for each group. All data presented are mean ± SEM, *P < 0.05 (unpaired *t*-test).

**Figure 2:**
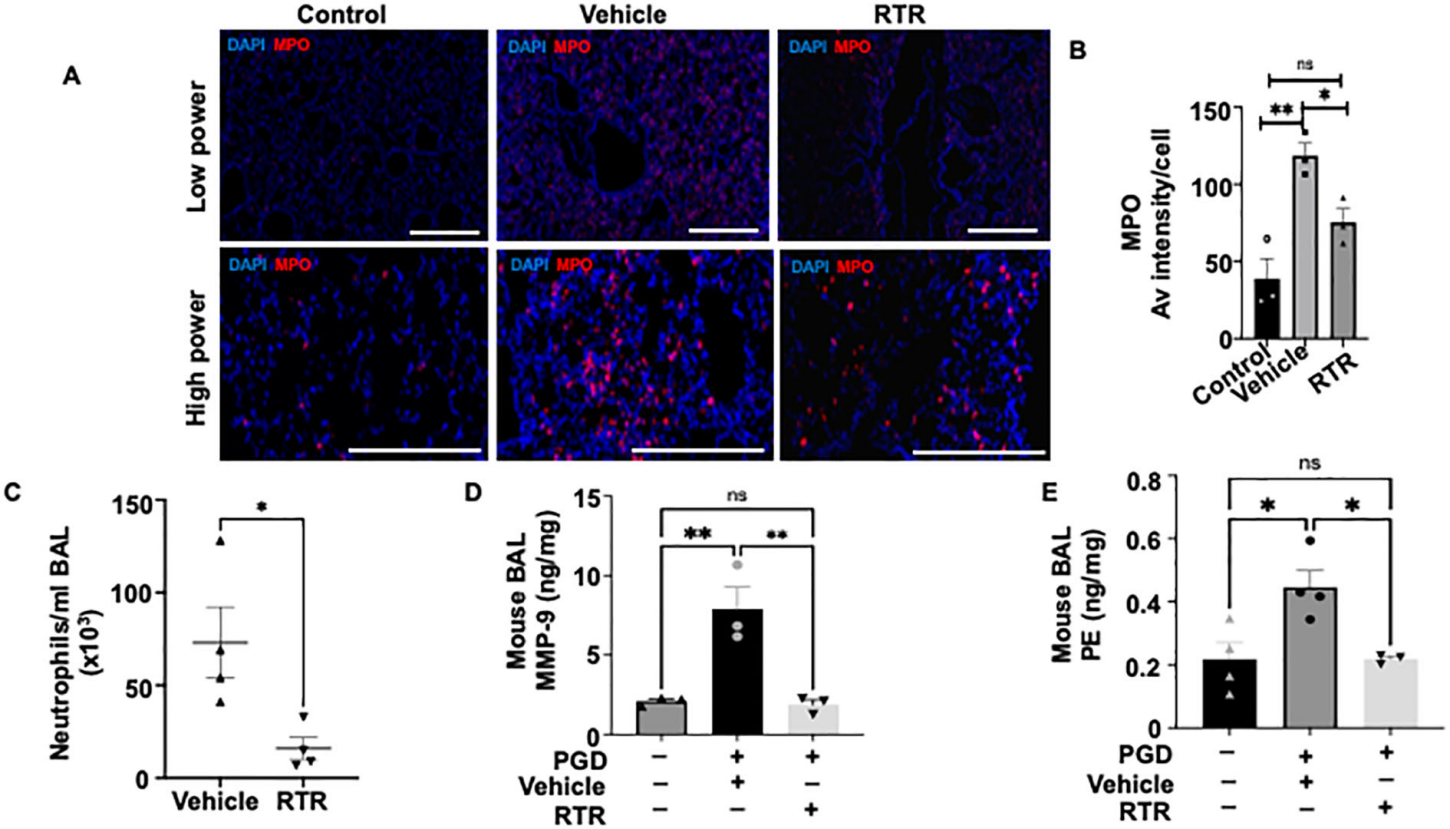
RTR reduces neutrophil recruitment and tends to ameliorate systemic inflammation in a Murine PGD Model. **(A)** Representative immunofluorescence images showing MPO staining (red) and nuclear staining with DAPI (blue) in control, vehicle-treated PGD, and RTR-treated PGD groups. **(B)** Average MPO immunofluorescence intensity per cell, demonstrating increased MPO signal in vehicle-treated PGD relative to controls and reduced MPO intensity in RTR-treated grafts. **(C) **Total neutrophil count in BAL fluid showing a significant reduction in neutrophils in RTR-treated PGD compared with vehicle-treated PGD. **(D) **Quantitative ELISA of MMP-9 in lung tissue lysates from control, vehicle-treated PGD, and RTR-treated PGD groups, demonstrating significant downregulation with RTR treatment. **(E)** Quantitative ELISA of PE in lung tissue lysates from control, vehicle-treated PGD, and RTR-treated PGD groups, showing a significant reduction in PE levels following RTR treatment. Data are presented as mean ± SEM; *P < 0.05 (unpaired t-test for two-sample comparison). Data are mean ± SEM; *P < 0.05, **P < 0.01 (one-way ANOVA with Tukey’s correction for three sample comparisons).

To determine whether the matrikine proline-glycine-proline (PGP) contributes to primary graft dysfunction (PGD), we examined specimens from our murine PGD model. Histology revealed extensive interstitial and perivascular neutrophilic inflammation, pleural thickening, and disruption of alveolar architecture in PGD allografts relative to controls (**Figure 1A**). To confirm the known pro-inflammatory phenotype of PGD, we conducted multiplex cytokine analysis of lung tissue lysates, which revealed a trend toward increased levels of inflammatory cytokines, including IL1α, IL1β, IP10, RANTES, and IL6, in PGD compared to controls (**Supplementary Figure 1**). Immunofluorescent staining for myeloperoxidase (MPO) confirmed a pronounced increase in neutrophil accumulation within PGD grafts (**Figures 2A, B**), underscoring neutrophil invasion as an early pathological feature. Because PGP arises during neutrophil-mediated extracellular-matrix degradation, we quantified its bioactive acetylated form (acPGP) in whole-lung lysates and found significantly higher levels in PGD allografts than in controls (**Figure 1B**). Our previous work showed that neutrophils are an inducible source of matrix metalloproteinase-9 (MMP-9) and prolyl endopeptidase (PE), enzymes essential for PGP generation. Consistent with this pathway, PE and MMP-9 were elevated in PGD lung lysates compared with controls (**Figures 2D, E**).

To confirm human clinical relevance, we measured PGP levels in bronchoalveolar lavage (BAL) fluid collected 72 hours after transplantation from recipients with grade 3 PGD (PGD3) and from non-PGD (PGD 0) controls. BAL PGP concentrations were markedly elevated in PGD samples (**Figure 1C**). To add rigor and to validate the pathway further, we utilized an independent human BAL cohort and found that BAL MMP9 and PE levels were significantly higher in the PGD3 specimens compared to PGD0 group (**Figures 1D, E**) paralleling the murine findings and implicating the PGP axis in human PGD.

### RTR-mediated PGP neutralization attenuates PGD severity in a murine model

**Figure 3:**
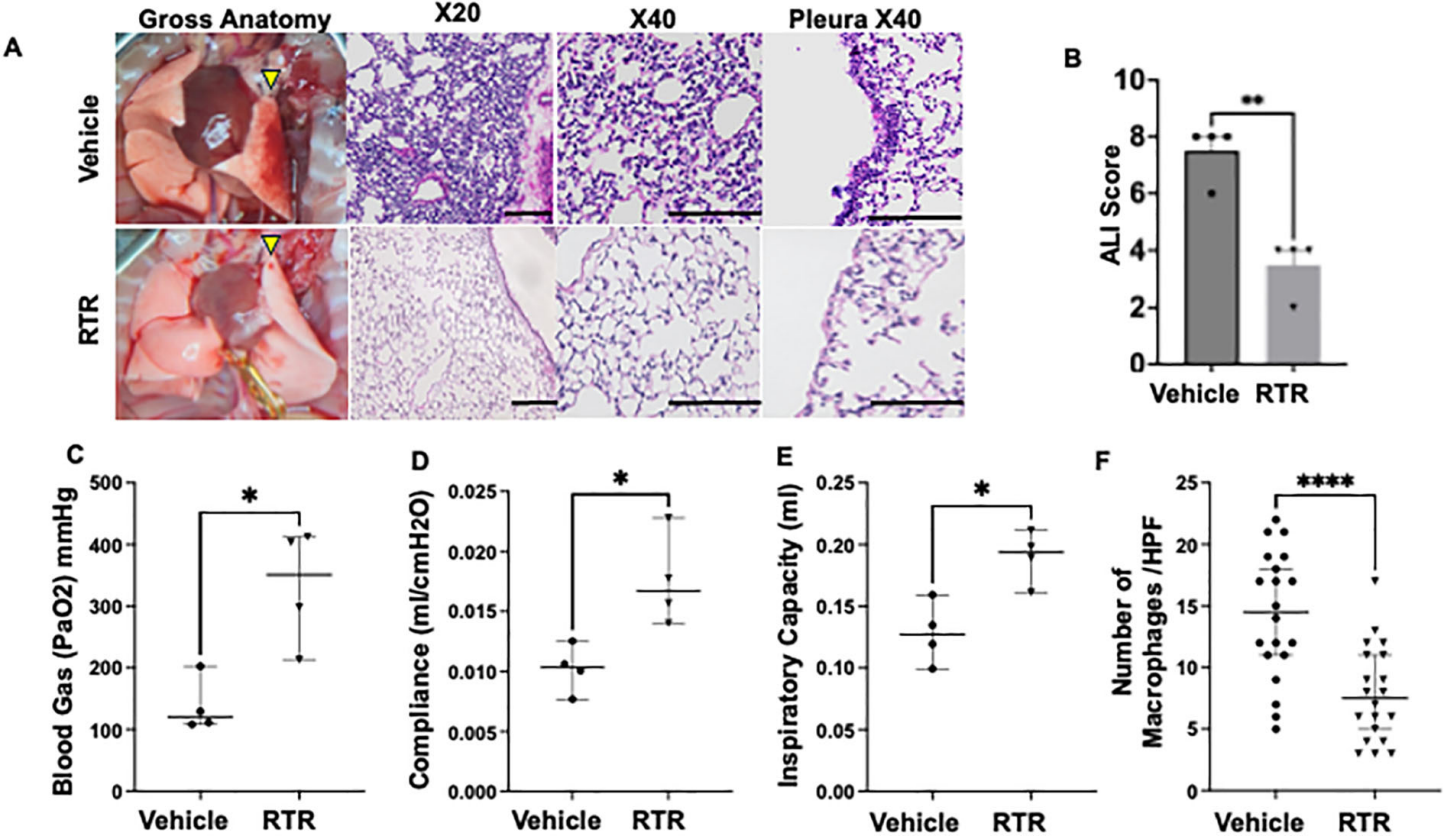
RTR-mediated PGP neutralization attenuates lung injury and improved graft function in a murine PGD model **(A)** Gross appearance of explanted lungs 4 h after reperfusion. Left panel: Vehicle-treated PGD grafts are edematous and erythematous, whereas RTR-treated allografts resemble controls (yellow arrowheads indicate the transplanted lungs). Right panel: H&E sections show reduced interstitial edema and inflammatory infiltrates in RTR-treated lungs. Scale bar = 100 µm. **(B)** Composite PGD injury scores (histology-based) for vehicle-treated PGD and RTR-treated PGD groups, mean ± SEM,**P < 0.01, unpaired T test. Sample size: n=4 for each group. **(C) **Oxygenation of the allograft based on arterial blood gas analysis. **(D, E)** Lung function parameters, including **(D)** compliance and **(E)** inspiratory capacity, are shown. **(F)** Quantitative analysis of macrophages in H&E-stained allograft sections. Data are presented as mean ± SEM. Sample size: n=4 for each group. *P < 0.05, **P < 0.01, ***P < 0.001, ****P < 0.0001; unpaired t test (2 sample comparison) or one-way ANOVA (3 sample comparison) with Tukey’s correction.

To evaluate whether pharmacologic blockade of PGP can attenuate PGD, we treated allografts with the PGP-neutralizing tripeptide L-arginine-threonine-arginine (RTR). Gross inspection after prolonged ischemia revealed visibly less edema and erythematous inflammation in RTR-treated grafts compared to the vehicle-treated PGD group (**Figure 3A**, left panel). Histology confirmed a significantly reduced neutrophil infiltration, interstitial edema (**Figure 3A**, right panel), and overall PGD injury scores in RTR-treated grafts (**Figure 3B**). Total protein concentration in BAL fluid in RTR-treated PGD mice tended to be lower compared with vehicle-treated PGD mice (**Supplementary Figure 2**). Oxygenation, as assessed by arterial blood gas analysis, and allograft lung function were significantly improved in the RTR-treated group compared with the vehicle group (**Figures 3C–E**). Further, H&E staining showed reduced infiltration of macrophage-shaped cells in the allografts of the RTR-treated group (**Figure 3F**). Immunofluorescent staining for myeloperoxidase (MPO) demonstrated lower signal intensity compared with vehicle-treated PGD grafts (**Figures 2A, B**), underscoring the attenuation of neutrophilic inflammation. Consistent with these findings, bronchoalveolar lavage showed a marked decrease in total neutrophil cell counts in RTR-treated lungs compared to the vehicle-treated PGD group (**Figure 2C**). Next, to investigate whether adaptive immunity was associated with the PGP forward feedback lung injury observed in PGD, we used a syngeneic (B6→B6) transplant model of PGD. Our results showed a similar attenuation of PGD in RTR-treated syngenic transplant compared to vehicle control (**Supplementary Figure 3**), indicating that the observed benefit from RTR was not dependent on adaptive alloimmunity.

### RTR disrupts the PGP-generating protease cascade in PGD

**Figure 4:**
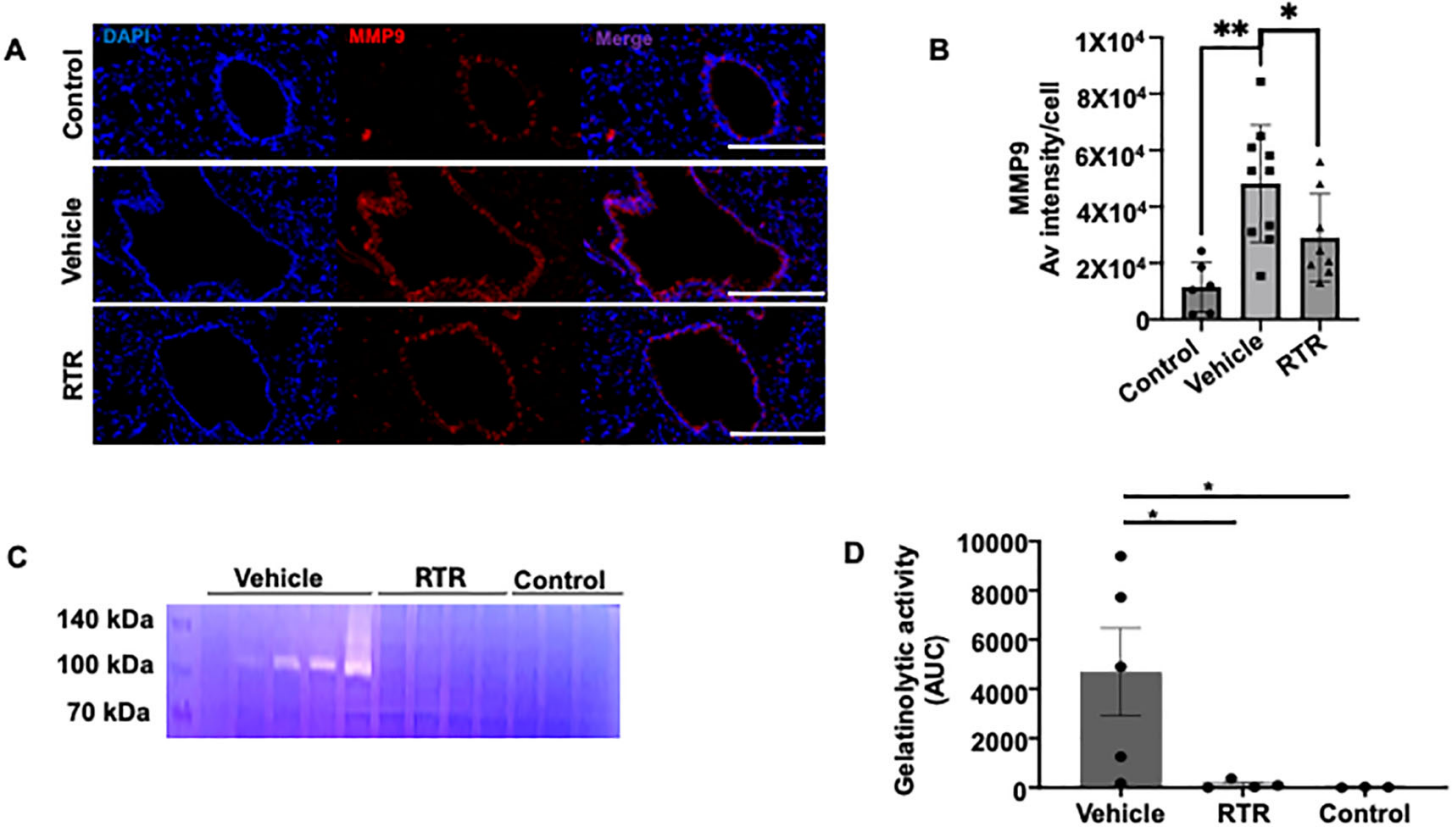
RTR reduces MMP-9 expression and enzymatic activity in PGD. **(A)** Representative immunofluorescence images showing MMP-9 staining (red) and nuclear staining with DAPI (blue) in control, vehicle-treated PGD, and RTR-treated PGD groups, demonstrating reduced MMP-9 protein expression in the RTR-treated PGD group. Scale bar = 50 µm. **(B)** Average MMP-9 immunofluorescence intensity per cell, showing increased MMP-9 signal in vehicle-treated PGD compared with controls and diminished MMP-9 intensity in RTR-treated grafts. Data in panels B are presented as mean ± SEM; *P < 0.05, **P < 0.01 (one-way ANOVA with Tukey’s correction) **(C)** Representative gelatin zymography illustrating bands corresponding to MMP-9 complexes or glycosylated form (~105 kDa) and a faint band for MMP-2 (~72 kDa). All samples were loaded with equal amounts of protein (54 μg per lane). **(D)** Quantification of gelatinolytic activity: band intensity was measured using ImageJ and expressed as the area under the curve (AUC). Data are presented as mean ± SEM; *P < 0.05 (Mann-Whitney test).

Because RTR attenuated both PGD severity and neutrophilia, we next interrogated its impact on the protease network that generates PGP. MMP-9 and PE are integral to generation of PGP. ELISA assays demonstrated that RTR treatment significantly reduced MMP-9 expression in PGD allografts compared to the vehicle-treated PGD group (**Figure 2D**). Likewise, Prolyl endopeptidase, required for PGP release, was down-regulated in RTR-treated mice (**Figure 2E**), suggesting that RTR intercepts the PGP-mediated feed-forward loop, thereby curbing further neutrophil recruitment and protease release. Importantly, RTR reduced both proteases to near-control levels. To add further rigor, we conducted MMP9 immunofloroscence which confirmed reduced MMP-9 expression with RTR treatment (**Figures 4A, B**). Next, we performed gelatin zymography to measure active MMP-9. Active MMP-9 levels were significantly lower in the RTR-treated group compared to vehicle controls (**Figures 4C, D**).

## Discussion

PGD remains the cause of early allograft injury, driving early mortality as well as subsequent chronic lung allograft dysfunction (3). Its histology is dominated by a massive neutrophil influx and microvascular leak (6, 17, 18); yet, the precise chemoattractant pathways contributing to these processes have remained elusive. Our study identifies the collagen-derived matrikine proline-glycine-proline (PGP) as the missing link and provides proof-of-concept that neutralizing PGP with the complementary tripeptide L-arginine–threonine–arginine (RTR) can blunt the entire cascade of neutrophil-mediated injury after lung transplantation providing a potential therapeutic target for alleviating PGD risk.

We first demonstrate that bioactive acetyl-PGP (acPGP) accumulates in mouse PGD allografts and in bronchoalveolar lavage (BAL) from human grade-3 PGD recipients, closely paralleling the neutrophil burden. This observation correlates with our earlier work showing elevated PGP in acute respiratory distress syndrome and post-transplant rejection (8, 9), suggesting that extracellular-matrix fragmentation is a shared driver of neutrophilic lung damage across pathological syndromes. These findings suggest that PGP may be a promising candidate biomarker for PGD detection. Prospective studies that serially measure PGP, from the time of reperfusion through the first 72 hours, will be needed to clarify its temporal kinetics and diagnostic utility.

Mechanistically, PGP liberation requires a two-step proteolytic pathway in which neutrophil-derived MMP-9 and the serine protease prolyl endopeptidase (PE) break down collagen into the tripeptide chemoattractant (11). Consistent with this pathway, both enzymes were upregulated in our PGD grafts, and importantly, returned to near-baseline levels when PGP was neutralized with RTR, indicating a reciprocal, feed-forward loop in which PGP recruits additional neutrophils that discharge more MMP-9 and PE. RTR peptide binds acPGP with high affinity, blocks CXCR2 engagement, and has previously reversed smoke-induced emphysema and chronic neutrophilic inflammation *in vivo (*13, 19). Here, a single intravenous dose delivered just before reperfusion reduced histological injury, BAL neutrophil counts, and composite PGD scores within four hours, an operative time window that is readily actionable in the operating room setting.

Our findings complement earlier work implicating neutrophil extracellular traps (NETs) and platelet activation in PGD pathogenesis (6, 17) but they extend the paradigm by identifying a specific matrikine/protease axis that is (i) measurable in clinical samples, (ii) upstream of multiple neutrophil effector functions, and (iii) druggable with a short, non-immunogenic peptide, RTR. Taken together, the data argue for prospective trials that pair peri-operative RTR administration with serial acPGP monitoring to stratify risk and guide therapy. They also raise the possibility of integrating RTR into *ex vivo* lung perfusion protocols, where peptide delivery and acPGP clearance could be titrated before implantation.

Limitations of our study include the short reperfusion window in our murine model, the small size of the human BAL cohort, the lack of longitudinal specimens, the lack of sex-matching in the cohorts, and the exclusively prophylactic dosing strategy. Future studies should investigate delayed or repeated RTR dosing, evaluate large-animal and *ex vivo* human-lung platforms, and explore interactions between the PGP axis and other injurious pathways, such as complement and NET formation. Likewise, further studies need to be conducted to assess the role of other chemoattractants and chemokines in PGD.

**Figure 5:**
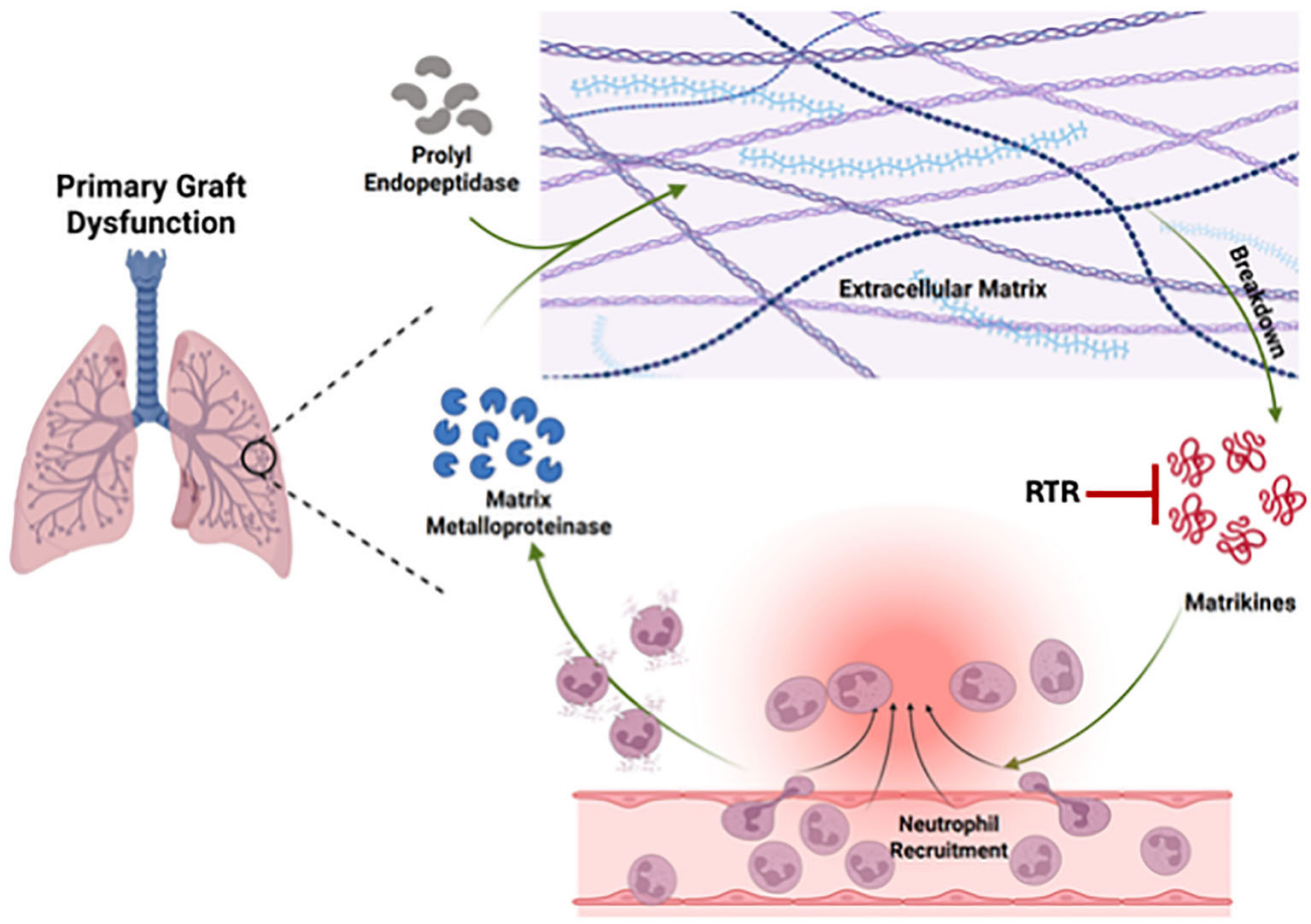
Schematic model of the PGP axis in PGD and its interruption by RTR.Ischemia–reperfusion injury triggers neutrophil recruitment and degranulation, releasing MMP-9 that cleaves extracellular-matrix collagen. PE trims these fragments to generate the neutrophil chemoattractant PGP, establishing a feed-forward loop of neutrophil influx, protease release, and vascular leak. The complementary tripeptide RTR binds acPGP, blunting neutrophil recruitment and secondarily reducing MMP-9/PE activity, thereby attenuating PGD severity.

In summary, we identify PGP as the molecular linchpin that couples extracellular matrix injury to sustained neutrophil recruitment in PGD and show that RTR-mediated PGP neutralization disrupts this feedforward circuit, restoring protease homeostasis and attenuating allograft injury (**Figure 5**).

## Data Availability

The original contributions presented in the study are included in the article/**Supplementary Material**, further inquiries can be directed to the corresponding author/s.
