# Supplementary figures and images for: Targeting the proline-glycine-proline-protease feed-forward loop attenuates primary graft dysfunction after lung transplantation
Source: Front Immunol. 2026 Feb 20;17:1655536. doi: 10.3389/fimmu.2026.1655536 (PMC12963002; doi:10.3389/fimmu.2026.1655536)

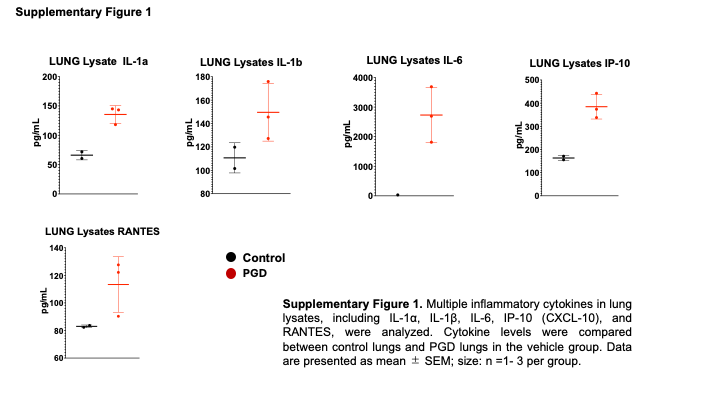

Supplement: Supplementary file 1 [file Image1.tiff]

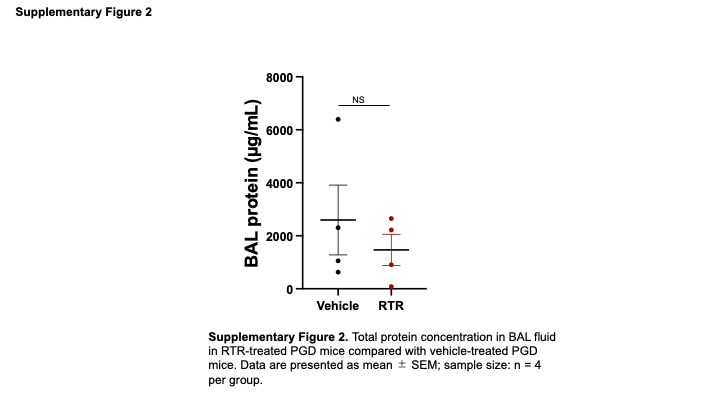

Supplement: Supplementary file 2 [file Image2.tiff]

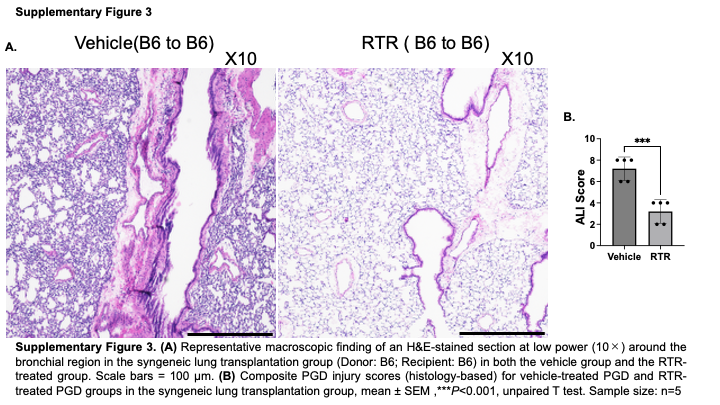

Supplement: Supplementary file 3 [file Image3.tiff]
