# Supplementary material for: Targeting the proline-glycine-proline-protease feed-forward loop attenuates primary graft dysfunction after lung transplantation
Source: Front Immunol. 2026 Feb 20;17:1655536. doi: 10.3389/fimmu.2026.1655536 (PMC12963002; doi:10.3389/fimmu.2026.1655536)
Supplement: Supplementary file 4 [file Table1.docx]

**Immunofluorescence**

Unstained slides were prepared from mouse tissue samples. The slides underwent deparaffinization and rehydration, followed by epitope retrieval using the IHC-TEK epitope retrieval steamer in accordance with standard protocols. Permeabilization was performed with 0.1% Triton X-100 in PBS. Subsequently, the slides were blocked with normal horse serum (Vector Laboratories, S-2000-20) for 3 hours at room temperature. After blocking, the tissue sections were incubated overnight at 4°C with primary antibodies diluted in normal horse serum; anti-H/M MPO (Catalog no. AF3667 R&D) and anti- Mouse MMP9 (Catalog no. ZK4559873 Invitrogen) respectively with dilutions of 1/500 each. The following day after 3 PBS washes, the sections were incubated for 60 minutes at room temperature with secondary antibodies diluted in the blocking buffer. The secondary antibodies used included: Alexa Fluor 594 donkey anti-mouse IgG (H+L) (Invitrogen, A-21203), and Alexa Fluor 647 donkey anti-goat IgG (H+L) (Invitrogen, AF394170).

Slides were mounted using VECTASHIELD® Antifade Mounting Medium with DAPI (Vector Laboratories), and fluorescent images were captured using the Flash Evident VS200 Slide Scanner at the Harvard Micron Microscopy Core. Image analysis and processing were carried out using FIJI-ImageJ software.

**ELISA**

**Mouse Lung Tissue and BAL Processing**

Sections of flash-frozen mouse lung tissue and mouse bronchoalveolar lavage (BAL) fluid were used for analysis of protease activity. Using a mortar and pestle chilled with liquid nitrogen, lung tissue was ground into a fine powder and resuspended in lysis buffer containing protease inhibitors to prevent degradation of target analytes. Tissue lysates were centrifuged at 10,000 rpm for 10 minutes to remove cellular debris, and the supernatants were collected and stored at −80 °C until analysis. BAL samples were similarly centrifuged before the immunoassay. Protein concentrations of tissue lysates were measured using a BCA assay and normalized accordingly. Samples were diluted in PBS to the desired concentration to ensure appropriate detection ranges for the ELISA. Total MMP-9 levels were quantified using the Mouse Total MMP-9 ELISA Kit (R&D Systems; Cat# MMPT9). Prolyl endopeptidase (PE) levels were measured using the Mouse PE ELISA Kit (MyBioSource; Cat# MBS9352431). All assays were performed according to the manufacturers’ instructions. After the final incubation and wash steps, absorbance was measured at 450 nm with a reference wavelength of 540 nm using a microplate reader.

**Human BAL ELISA**

Human bronchoalveolar lavage (BAL) fluid was collected during clinically indicated procedures and immediately placed on ice. Samples were passed through sterile gauze or a 70-µm cell strainer to remove mucus and debris, then centrifuged at 400 × g for 10 minutes at 4 °C. The cell-free supernatant was collected, aliquoted to avoid repeated freeze–thaw cycles, and stored at −80 °C until ELISA analysis. Human ELISA kits for MMP-9 (Abcam, ab246539) and prolyl endopeptidase (PE; Abcam, ab193701) were used according to the manufacturers’ instructions. Standard curves were generated from known concentrations, and analyte levels were calculated from the mean of duplicate measurements.

**Mouse Lung Lysate Cytokine Analysis**

Lung tissue wes homogenized in RIPA buffer supplemented with Complete Proteinase Inhibitor Cocktail (Millipore Sigma). Total tissue proteins were quantified using a Pierce BCA protein assay kit (Thermo Scientific). The concentration of IL-1α, IL-1β, IL-6, IP-10, RANTES, were measured by multiplex cytokine analysis (Merck Millipore MCYT1-190K) according to the manufacturer’s instructions.
